# Goserelin (Zoladex™) – its role in early breast cancer in pre- and perimenopausal women

**DOI:** 10.1054/bjoc.2001.1981

**Published:** 2001-02-01

**Authors:** W Jonat

**Affiliations:** Gynaecology and Obstetrics Clinic, University of Kiel, D-24105 Kiel, Germany

**Keywords:** adjuvant, early breast cancer, ovarian ablation, premenopausal, goserelin, Zoladex

## Abstract

Current standard adjuvant therapies for premenopausal women with early breast cancer include ovarian ablation by surgery or irradiation, chemotherapy and tamoxifen. The value of ovarian ablation in prolonging the survival of premenopausal patients with early breast cancer was clearly established by the analyses performed by the Early Breast Cancer Trialists' Collaborative Group in 1996. More recently, the value of ovarian suppression using the luteinizing hormone releasing hormone analogue goserelin as adjuvant therapy in pre-/perimenopausal women with early breast cancer has been confirmed in a series of studies involving over 8000 patients. The results from these studies provide evidence that goserelin, alone or in combination with tamoxifen, is at least as effective as cytotoxic chemotherapy in patients with hormone receptor-positive tumours and is effective when used after adjuvant chemotherapy. The use of goserelin in the management of early breast cancer presents an option which can avoid the side-effects experienced with cytotoxic chemotherapy and may offer unique benefits to premenopausal patients. The consolidation of these emerging results should help in defining the optimal role for goserelin in pre-/perimenopausal patients with early breast cancer. © 2001 Cancer Research Campaign


*British Journal of Cancer* (2001) **85**(Suppl 2), 1–5
© 2001 Cancer Research Campaign
doi: 10.1054/ bjoc.2001.1981, available online at http://www.idealibrary.com on IDEAL

### Goserelin (Zoladex™) – its role in early breast cancer in pre- and perimenopausal women

**W Jonat**

Gynaecology and Obstetrics Clinic, University of Kiel, D-24105 Kiel, Germany

**Summary** Current standard adjuvant therapies for premenopausal women with early breast cancer include ovarian ablation by surgery or irradiation, chemotherapy and tamoxifen. The value of ovarian ablation in prolonging the survival of premenopausal patients with early breast cancer was clearly established by the analyses performed by the Early Breast Cancer Trialists' Collaborative Group in 1996. More recently, the value of ovarian suppression using the luteinizing hormone releasing hormone analogue goserelin as adjuvant therapy in pre-/perimenopausal women with early breast cancer has been confirmed in a series of studies involving over 8000 patients. The results from these studies provide evidence that goserelin, alone or in combination with tamoxifen, is at least as effective as cytotoxic chemotherapy in patients with hormone receptor-positive tumours and is effective when used after adjuvant chemotherapy. The use of goserelin in the management of early breast cancer presents an option which can avoid the side-effects experienced with cytotoxic chemotherapy and may offer unique benefits to premenopausal patients. The consolidation of these emerging results should help in defining the optimal role for goserelin in pre-/perimenopausal patients with early breast cancer. © 2001 Cancer Research Campaign.

**Keywords**: adjuvant; early breast cancer; ovarian ablation; premenopausal; goserelin; Zoladex

### INTRODUCTION

Systemic adjuvant therapies following surgical removal of the primary tumour are advocated in patients with early breast cancer with the aim of eliminating or reducing the incidence of distant metastases, ultimately prolonging disease-free survival (DFS) and overall survival (OS). The presence of endogenous oestradiol plays a significant role in the progression of disease and its impact is largely dependent on the hormone receptor status of the tumour. Approximately 60% of pre-/perimenopausal patients with primary breast cancer have oestrogen-receptor (ER)-positive tumours (Pujol et al, 1998; Aebi et al, 2000; Kroman et al, 2000). Thus, removal of the source of endogenous oestrogen in premenopausal patients is likely to prevent growth of tumours that are sensitive to circulating oestrogens.

The value of adjuvant ovarian ablation has long been recognized as a valuable treatment option for premenopausal women with early breast cancer. This was confirmed in 1996 by the Early Breast Cancer Trialists' Collaborative Group (EBCTCG, 1996). In reviewing data from adjuvant trials, they found that in 2102 women under 50 years-of-age with node-negative or node-positive early breast cancer, ablation of functioning ovaries by surgical oophorectomy or radiotherapy significantly improved 15-year disease-free and overall survival compared with controls. In direct (Scottish Cancer Trials Breast Group and ICRF Breast Unit, 1993; Ejlertsen et al, 1999) and indirect (EBCTCG, 1992) comparisons, ovarian ablation has been shown to have comparable efficacy to chemotherapy. Despite the efficacy of ovarian ablation by surgery or irradiation in prolonging survival, they generally induce a permanent menopause with associated long-term effects, such as loss of bone mineral density (BMD) and increased risk for cardiovascular problems (Goodwin et al, 1999; Pfeilschifter and Diel, 2000). Growing evidence indicates that there may be additional benefits offered by medical endocrine therapies which induce a reversible state of ovarian suppression.

Over the past decade, the luteinizing hormone releasing hormone (LHRH) analogue goserelin (Zoladex™) alone (Blamey et al, 1992; 1993), or with tamoxifen (Jonat et al, 1995), has proven effective for the treatment of advanced (metastatic or locally advanced) breast cancer in pre-/perimenopausal women resulting in objective response rates similar to oophorectomy (Boccardo et al, 1994; Taylor et al, 1998). This success of goserelin in treating metastatic disease, and more recently the growing evidence from ongoing international trials of goserelin in early disease, indicate that goserelin may provide a valuable alternative to chemotherapy for premenopausal women with hormone receptor-positive early breast cancer.

### MODE OF ACTION OF GOSERELIN

LHRH analogues, such as goserelin, reduce circulating concentrations of oestrogen in premenopausal women via an inhibitory effect on the hypothalamic–pituitary–ovarian axis. At the cellular level, LHRH analogues bind to LHRH receptors on pituitary gland cells, an action which causes an initial surge in the secretion of luteinizing hormone (LH). Once bound to ligand, these LHRH receptors form clusters, which are then sequestered within the cell, thereby reducing the number of unoccupied LHRH receptors. These unoccupied receptors are maintained at low levels by the presence of the LHRH analogue, ultimately resulting in reduced LH secretion. In turn, the reduced plasma LH causes a decrease in circulatory oestradiol (the main source of oestrogen in premenopausal women) to levels comparable to the postmenopausal state within 21 days, which are maintained with continued administration of LHRH analogues (Furr, 1989).

### ADJUVANT TREATMENT OPTIONS IN PRE-/PERIMENOPAUSAL PATIENTS

Current adjuvant therapies for the pre-/perimenopausal patient with early breast cancer include: ovarian ablation or medical ovarian suppression with or without tamoxifen; tamoxifen alone; or postoperative chemotherapy with or without tamoxifen.

The choice of adjuvant therapy for pre- and perimenopausal women with early breast cancer is heavily influenced by prognostic and predictive factors. Such factors include: axillary lymph node status, tumour size, histological or nuclear grade of the tumour, menopausal status and hormone-receptor status. The hormone-receptor status of the tumour – both ER and progesterone receptor (PgR) – is highly predictive of the response to endocrine therapies (EBCTCG, 1998). In particular, patients with tumours expressing ER and PgR tend to show a greater response rate and prolonged DFS and overall survival when treated with endocrine therapies (Clark, 1999; EBCTCG, 1998). Consequently, there is a strong case for trying to identify those patients who are most likely to respond favourably to adjuvant endocrine treatment. Ovarian ablation achieved by LHRH analogue therapy may be an important treatment option in these patients, offering a targeted, potentially reversible ovarian suppression that may avoid the long-term effects associated with premature menopause.

### UPDATE OF GOSERELIN IN CLINICAL TRIALS

A number of ongoing trials involving pre-/perimenopausal women with early breast cancer are now reporting on the efficacy and tolerability of goserelin, either alone or in combination with tamoxifen, and as an alternative or in addition to standard chemotherapy regimens (Figure 1). This programme of trials was designed to investigate the use of goserelin in different settings in order to define more clearly the future role of goserelin in early breast cancer.

### Goserelin monotherapy

#### *'Zoladex' Early Breast Cancer Research Association (ZEBRA) trial*

The ZEBRA trial represents the first direct comparison of adjuvant goserelin monotherapy with chemotherapy in pre-/perimenopausal patients aged 50 years or less with node-positive early breast cancer. This large (*n* = 1640), randomized, multicentre (102 centres) trial assessed the effect of these treatments on DFS, OS and the side-effect profile associated with each treatment. The ZEBRA trial protocol also included sub-studies to assess quality of life (QoL) and BMD.

The ZEBRA study reported that goserelin (3.6 mg every 28 days for 2 years) was equivalent to cyclophosphamide/methotrexate/5-fluorouracil (CMF, 6 × 28-day cycles) for DFS in ER-positive patients after a median follow-up of 6 years (HR = 1.01, 95% CI = 0.84–1.20) (Kaufmann, 2001). The patients with ER-positive tumours accounted for approximately 74% of the study population. However, for ER-negative patients (approximately 19% of the study population), there was a significant advantage in favour of CMF for DFS (HR = 1.76, 95% CI = 1.27–2.44). The onset of amenorrhoea occurred more rapidly with goserelin treatment than with CMF, with over 95% of patients receiving goserelin achieving amenorrhoea by 6 months, compared with approximately 60% of patients in the CMF group. Furthermore, amenorrhoea was found to be reversible in the majority of patients receiving goserelin but permanent with CMF: only 23% of goserelin patients remained amenorrhoeic at 3 years compared with 77% in the CMF group (Jonat, 2000).

In terms of tolerability to therapy, typical side-effects of chemotherapy (e.g. alopecia, nausea and vomiting, and infection) were substantially higher in patients receiving CMF during the 6-month CMF treatment period. Menopausal symptoms (e.g. vaginal dryness and hot flushes) were initially lower in the CMF group compared with the goserelin group. However, these endocrine-

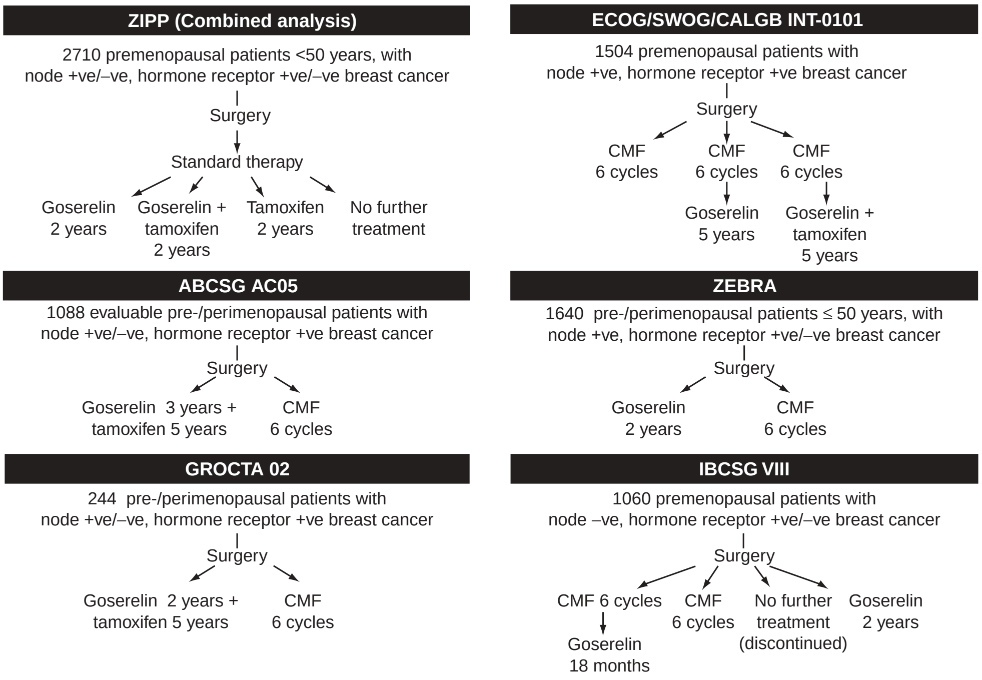

**Figure 1** Clinical trials designs for studies involved in the evaluation of goserelin as adjuvant treatment in early breast cancer in pre-/perimenopausal patients

 © 2001 Cancer Research Campaign related side-effects remained virtually unchanged in the CMF group during follow-up, whereas 1 year after the cessation of goserelin the incidence of these effects decreased to below that in the CMF group (Jonat, 2000).

In total, 514 patients receiving goserelin and 496 patients receiving CMF in 86 centres were included in the QoL sub-study. The improvement from baseline in overall QoL score was significantly ($P$ < 0.0001) greater in the goserelin-treated group compared with the CMF-treated group during the first 3–6 months. However, at 1, 2 and 3 years there were no significant differences in overall QoL scores between the two treatment groups (de Haes, 2001).

In the BMD sub-study, eight centres contributed data from 96 selected patients. Losses in BMD were observed for both treatment groups during the first 2 years of the study, with the losses being greater in the goserelin group. However, at 3 years (1 year after cessation of goserelin therapy), partial recovery of BMD was seen in the goserelin group, whereas losses persisted in the CMF group overall throughout follow-up. As a result, no significant differences in BMD were observed between the two treatment groups at the 3-year assessment. In addition, the observed changes in BMD levels appeared to be related to menstrual status in both treatment groups (Fogelman, 2001).

### Goserelin plus tamoxifen

### *The Austrian Breast Cancer Study Group (ABCSG) ACO5 trial*

The ABCSG trial was designed to compare ovarian suppression with goserelin plus tamoxifen versus CMF in premenopausal patients with ER-positive/PgR-positive, node-positive/node-negative breast cancer (Jakesz et al, 2001). At a median follow-up of 50 months, results from 1088 evaluable patients showed a significantly improved recurrence-free survival for goserelin and tamoxifen combination therapy compared with CMF therapy ($P$ < 0.02).

### *Italian Breast Cancer Adjuvant Study Group (GROCTA 02) trial*

The GROCTA 02 trial was a randomized trial designed to compare the efficacy of chemotherapy (CMF, $n$ = 120) with that of tamoxifen plus ovarian suppression (oophorectomy $n$ = 6, ovarian irradiation $n$ = 31, goserelin $n$ = 87) in 244 pre-/perimenopausal patients with node-positive/node-negative, ER-positive breast cancer (Boccardo et al, 2000; 2001). Results at a median follow-up of 89 months revealed that tamoxifen plus ovarian suppression achieved comparable results in terms of DFS and OS to those of CMF, regardless of nodal status. Furthermore, there was no difference in clinical outcome of patients treated with oophorectomy or ovarian irradiation compared with those treated with goserelin.

### Goserelin in addition to standard therapy

### *Zoladex in Premenopausal Patients (ZIPP) trial*

The ZIPP trial was designed to investigate the effect of adding goserelin to standard therapy (surgery ± radiotherapy ± cytotoxic chemotherapy ± tamoxifen) in premenopausal patients with early breast cancer, regardless of nodal or tumour ER status. Patients were randomized to receive goserelin, tamoxifen, goserelin plus tamoxifen or no further treatment. Of the 2710 patients randomized, 2032 were of known tumour ER status and of these 68% were ER-positive. Patients who received goserelin in addition to standard therapy showed a significant improvement in recurrence-free survival (RR = 0.80, 95% CI = 0.70–0.92, $P$ < 0.001) and overall survival (RR = 0.82, 95% CI = 0.67–0.99, $P$ = 0.04) compared with those who did not receive goserelin (Baum et al, 2001).

### Goserelin alone or combined with tamoxifen following cytotoxic chemotherapy

### *Eastern Cooperative Oncology Group (ECOG)/South Western Oncology Group (SWOG)/Cancer and Leukemia Group B (CALGB) – INT-0101 trial*

This trial was instigated to assess the use of goserelin with or without tamoxifen following chemotherapy. In 1504 eligible premenopausal patients with node-positive, hormone receptor-positive breast cancer, patients receiving six cycles of cyclophosphamide/adriamycin/5-fluorouracil (CAF) were compared with patients receiving CAF followed by goserelin (Z) either alone (CAFZ) or with tamoxifen (CAFZT) (Davidson et al, 1999). There was a significant ($P$ < 0.01) improvement in 5-year DFS for CAFZT-treated patients (77%) compared with patients treated with CAFZ (70%), with a trend towards improved 5-year DFS for CAFZ versus CAF (67%).

### Goserelin alone or in combination with cytotoxic chemotherapy

### *International Breast Cancer Study Group (IBCSG) VIII trial*

In the IBCSG VIII trial, 1060 premenopausal patients with node-negative, hormone receptor-positive/negative early breast cancer were initially randomized to CMF followed by goserelin, CMF alone, goserelin alone or no treatment. The no-treatment arm was

**Table 1** Summary of key results to date from the goserelin adjuvant trials programme involving pre-/perimenopausal patients with early breast cancer

| Trial | Key results |
|---|---|
| ZEBRA | Goserelin demonstrates equivalent efficacy to CMF in patients with ER-positive tumours but without the distressing side-effects of cytotoxic chemotherapy |
| ZEBRA | Amenorrhoea and associated side-effects are reversible with goserelin in the majority of patients but appear to be permanent with CMF |
| GROCTA 02 and ABCSG AC05 | Goserelin combined with tamoxifen is at least as effective as CMF in patients with hormone receptor-positive tumours |
| ZIPP | Addition of goserelin to standard therapy (± radiotherapy ± chemotherapy ± tamoxifen) is beneficial |
| INT-0101 | Addition of goserelin, alone or in combination with tamoxifen, to CAF therapy is beneficial in patients with hormone receptor-positive tumours |
| IBCSG VIII | Adjuvant therapy in node-negative, premenopausal patients improves outcome compared with no adjuvant therapy |

© 2001 Cancer Research Campaign subsequently dropped because evidence from other trials showed that adjuvant treatment improved outcome in this patient population. An analysis of the effect of adjuvant treatment compared with no adjuvant treatment in this trial has confirmed that adjuvant treatment improves outcome in premenopausal patients with node-negative early breast cancer (Castiglione-Gertsch et al, 2000). Further results from this trial are awaited.

### TRIALS SUMMARY

A summary of the key findings from trials involving goserelin for the treatment of pre-/perimenopausal patients with early breast cancer is shown in Table 1. The ZEBRA trial is the first report of a direct comparison of LHRH analogue monotherapy with cytotoxic chemotherapy following initial removal of the primary tumour by surgery (± radiotherapy) in pre-/perimenopausal patients with early breast cancer. The results from this trial show that, in this patient population, goserelin demonstrates equivalent efficacy to CMF in patients with ER-positive tumours. The randomized trials discussed in this review support the use of goserelin alone or combined with tamoxifen and/or cytotoxic chemotherapy or integrated into treatment strategies following standard therapy to provide a beneficial effect in premenopausal patients with hormone receptor-positive early breast cancer.

### CONCLUSIONS

Ovarian suppression undoubtedly provides a valuable treatment option in premenopausal patients with hormone receptor-positive tumours. Goserelin provides an alternative to cytotoxic chemotherapy without the associated distressing side-effects. Consequently, the addition of goserelin to the adjuvant treatment armamentarium further extends the choice of treatments available to premenopausal women with hormone receptor-positive breast cancer. It provides the opportunity of a temporary, rather than permanent, ovarian ablation, without loss of efficacy when compared with CMF treatment. Moreover, goserelin offers a means of avoiding cytotoxic chemotherapy which may be a requirement for some patients, and also a treatment which, in some patients, may be combined with tamoxifen and/or cytotoxic chemotherapy, with beneficial results.

The benefits of a temporary ablation in premenopausal patients should not be underestimated; not only does it avoid the long-term consequences on, for example, the cardiovascular system and BMD, it also offers this patient group a choice which avoids the need for cytotoxic chemotherapy. In addition, hormone receptor status clearly plays an important role in treatment outcome with endocrine therapies and these studies confirm the need for routine diagnosis of hormone receptor status in the management of early breast cancer, to identify patients most likely to benefit from endocrine therapies.

### REFERENCES

Aebi S, Gelber S, Castiglione-Gertsch M, Gelber RD, Collins J, Thurlimann B, Rudenstam CM, Lindtner J, Crivellari D, Cortes-Funes H, Simoneini E, Werner ID, Coates AS and Goldhirsch A (2000) Is chemotherapy alone adequate for young women with oestrogen-receptor-positive breast cancer? *Lancet* **355**: 1869–1874

Baum M, Houghton J, Odling-Smee W, Rutqvist LE, Fornander T, Nordenskjold B, Nicolucci A and Mari E (2001) Adjuvant 'Zoladex' in premenopausal patients with early breast cancer: results from the ZIPP trial. *Breast* **10**(Suppl 1): S32–S33 (Abst P64)

Blamey RW, Jonat W, Kaufmann M, Bianco AR and Namer M (1992) Goserelin depot in the treatment of premenopausal advanced breast cancer. *Eur J Cancer* **28A**: 810–814

Blamey RW, Jonat W, Kaufmann M, Bianco AR and Namer M (1993) Survival data relating to the use of goserelin depot in the treatment of premenopausal advanced breast cancer. *Eur J Cancer* **29A**: 1498

Boccardo F, Rubagotti A, Perrotta A, Amoroso D, Balestrero M, De Matteis A, Zola P, Sismondi P, Francini G and Petrioli R (1994) Ovarian ablation versus goserelin with or without tamoxifen in pre-/perimenopausal patients with advanced breast cancer: results of a multicentric Italian study. *Ann Oncol* **5**: 337–342

Boccardo F, Rubagotti A, Amoroso D, Mesiti M, Romeo D, Sismondi P, Giai M, Genta F, Pacini P, Distante V, Bolognesi A, Aldrighetti D and Farris A (2000) Cyclophosphamide, methotrexate, and fluorouracil versus tamoxifen plus ovarian suppression as adjuvant treatment of estrogen receptor-positive pre-/perimenopausal breast cancer patients: results of the Italian Breast Cancer Adjuvant Study Group 02 randomized trial. *J Clin Oncol* **18**: 2718–2727

Boccardo F, Rubagotti A, Amoroso D, Mesiti M, Romeo D, Aldrighetti D, Genta F, Sismondi P, Irtelli L, Donati D, Farris A and Schieppati G (2001) CMF vs tamoxifen (TAM) plus ovarian suppression (OS) as adjuvant treatment of ER positive (ER +) pre/perimenopausal breast cancer (BCA) patients (pts). *Breast* **10**(Suppl 1): S32 (Abst P62)

Castiglione-Gertsch M, Gelber RD, O'Neill A, Coates AS and Goldhirsch A (2000) Systemic adjuvant treatment for premenopausal node-negative breast cancer. *Eur J Cancer* **36**: 549–550

Clark GM (1999) Prognostic and predictive factors for primary breast cancer. *Proc ASCO* **Educational Book**: 205–207

Davidson NE, O'Neill A, Vukov A, Osborne CK, Martino S, White D and Abeloff MD (1999) Effect of chemohormonal therapy in premenopausal, node-positive, receptor-positive breast cancer: an Eastern Cooperative Oncology Group Phase III Intergroup Trial (E5188, INT-0101). *Breast* **8**: 232–233 (Abst 69)

de Haes H (2001) Comparison of quality of life in pre-/perimenopausal patients treated with Zoladex™ or CMF as adjuvant therapy for the management of node-positive early breast cancer: results from the ZEBRA study. *Breast* **10** (Suppl 1): S32 (Abst P61)

Early Breast Cancer Trialists' Collaborative Group (1992) Systemic treatment of early breast cancer by hormonal, cytotoxic or immune therapy. 133 randomized trials involving 31,000 recurrences and 24,000 deaths among 75,000 women. *Lancet* **339**: 1–15, 71–85

Early Breast Cancer Trialists' Collaborative Group (1996) Ovarian ablation in early breast cancer: overview of the randomised trials. *Lancet* **348**: 1189–1196

Early Breast Cancer Trialists' Collaborative Group (1998) Tamoxifen for early breast cancer: an overview of the randomised trials. *Lancet* **351**: 1451–1467

Ejlertsen B, Dombernowsky P, Mouridsen HT, Kamby C, Kjaer M, Rose C, Andersen KW, Jensen B, Bengtsson NO and Bergh J (1999) Comparable effect of ovarian ablation (OA) and CMF chemotherapy in premenopausal hormone receptor positive breast cancer patients (PRP). *Proc ASCO* **18**: 66a (Abstr 248)

Fogelman I (2001) Assessment of bone mineral density in pre-/perimenopausal women treated with Zoladex™ versus CMF adjuvant therapy for management of node-positive, early breast cancer: results from the ZEBRA study. *Breast* **10** (Suppl. 1): S31 (Abstr P59)

Furr BJA (1989) Pharmacology of the luteinising hormone-releasing hormone (LHRH) analogue, Zoladex. *Horm Res* **32**: 86–92

Goodwin PJ, Ennis M, Pritchard KI, Trudeau M and Hood N (1999) Risk of menopause during the first year after breast cancer diagnosis. *J Clin Oncol* **17**: 2365–2370

Jakesz R, Hausmaninger H, Samonigg H, Kubista E, Gnant C, Menzel T, Bauernhofer T, Seifert M, Haider K, Steger G, Mlineritsch B, Steindorfer P, Kwasny, Fridrik M and Wette V (2001) Complete endocrine blockade with tamoxifen and goserelin is superior to CMF in the adjuvant treatment of premenopausal, lymph node-positive and -negative patients with hormone-responsive breast cancer. *Breast* **10**(Suppl 1): S10 (Abst S26)

Jonat W, Kaufmann M, Blamey RW, Howell A, Collins JP, Coates A, Eiermann W, Janicke F, Njordenskold B, Forbes JF and Kolvenbag GJCM (1995). A randomised study to compare the effect of the luteinising hormone releasing hormone (LHRH) analogue goserelin with or without tamoxifen in pre-and perimenopausal patients with advanced breast cancer. *Eur J Cancer* **31A**: 137–142

*© 2001 Cancer Research Campaign*

Jonat W (2000) 'Zoladex' versus CMF adjuvant therapy in pre-/perimenopausal breast cancer: tolerability and amenorrhoea comparisons. *Proc ASCO* **19**: 87a (Abst 333)

Kaufmann M (2001) Zoladex™ (goserelin) vs CMF as adjuvant therapy in pre-/perimenopausal, node-positive early breast cancer: preliminary efficacy results from the ZEBRA study. *Breast* **10**(Suppl 1): S30 (Abst P53)

Kroman N, Jensen MB, Wohlfahrt J, Mourisden HT, Andersen PK and Melbye M (2000) Factors influencing the effect of age on prognosis in breast cancer: population-based study. *BMJ* **320**: 474–479

Pfeilschifter J and Diel IJ (2000) Osteoporosis due to cancer treatment: pathogenesis and management. *J Clin Oncol* **18**: 1570–1593

Pujol P, Daures JP, Thezenas S, Guilleux F, Rouanet P and Grenier J (1998) Changing estrogen and progesterone receptor patterns in breast carcinoma during the menstrual cycle and menopause. *Cancer* **83**: 698–705

Scottish Cancer Trials Breast Group and ICRF Breast Unit (1993) Adjuvant ovarian ablation versus CMF chemotherapy in premenopausal women with pathological stage II breast carcinoma: the Scottish trial. *Lancet* **341**: 1293–1298

Taylor CW, Green S, Dalton WS, Martino S, Rector D, Ingle JN, Robert NJ, Budd GT, Paradelo JC, Natale RB, Bearden JD, Mailliard JA and Osborne CK (1998) Multicenter randomized clinical trial of goserelin versus surgical ovariectomy in premenopausal patients with receptor-positive metastatic breast cancer: an Intergroup Study. *J Clin Oncol* **16**: 994–999

*© 2001 Cancer Research Campaign*
