# Goserelin (Zoladex™) – its role in early breast cancer in pre- and perimenopausal women

**DOI:** 10.1038/sj.bjc.6600652

**Published:** 2002-12-02

**Authors:** W Jonat

## Abstract

*British Journal of Cancer* (2002) **87**, 1480–1480. doi:10.1038/sj.bjc.6600652 www.bjcancer.com

© 2002 Cancer Research UK

**Correction to:** *British Journal of Cancer* 2001; **85** (Suppl 2): 1–5. doi:10.1054/bjoc.2001.1981

## 

An error has been noted within Figure 1

**Figure 1:**
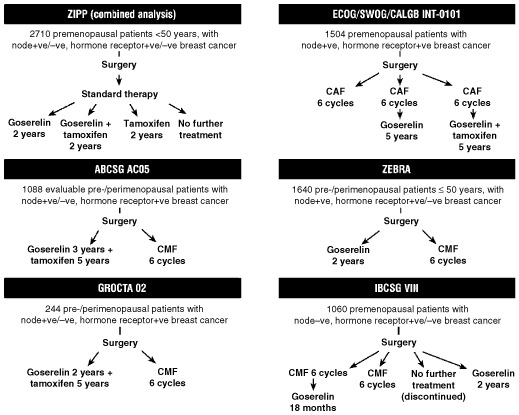
Clinical trials designs for studies involved in the evaluation of goserelin as adjuvant treatment in early breast cancer in pre-/perimenopausal patients.

. The chemotherapy regimen shown in the ECOG/SWOG/CALGB INT-0101 study is incorrect and should be six cycles of CAF (cyclophosphamide/adriamycin/5-fluorouracil) and not six cycles of CMF (cyclophosphamide/methotrexate/5-fluorouracil), as presented. The corrected version of the figure is shown here.

